# Predictors of attendance to group exercise: a cohort study of older adults in long-term care facilities

**DOI:** 10.1186/s12877-015-0043-y

**Published:** 2015-04-02

**Authors:** Susanne Finnegan, Julie Bruce, Sarah E Lamb, Frances Griffiths

**Affiliations:** Warwick Clinical Trials Unit, Division of Health Sciences, University of Warwick, Coventry, CV4 7AL UK; Oxford Clinical Trials Research Unit, John Radcliffe Hospital, Oxford, OX3 9DU UK

**Keywords:** Exercise, Physical activity, Older people, Attendance, Participation, Long-term care

## Abstract

**Background:**

The benefits of regular exercise and physical activity are well known. Those living in long-term care are often frail, but have the potential to benefit from physical activity; yet are less likely to be offered exercise. Promoting exercise within residential and nursing homes may reduce health risks associated with inactivity in this population. The aim of this cohort study is to identify predictors of attendance at physiotherapy led exercise groups offered to older adults residing in long-term care in the United Kingdom.

**Methods:**

One thousand and twenty three older adults residing in residential and nursing homes, were recruited to the Older People's Exercise in Residential and nursing Accommodation (OPERA) cluster randomised controlled trial. Secondary analysis of 428 adults (aged 75 to 107) randomised to twice-weekly physiotherapy-led group exercise sessions for 12 months was undertaken. Using attendance data, linear regression analysis was utilised to separately identify individual and home-level factors predictive of attendance at exercise in the residential and nursing homes.

**Results:**

Of 428 older adults, 326 lived in residential homes and 102 in nursing homes. Mean age of the sample was 88.0 years and the majority of residents were female (324/428, 76%). Pre-intervention assessment suggested that most residents had moderate cognitive impairment; median (range) Mini Mental State Examination scores in residential homes were 19 (0–30) and 14 (0–29) in nursing homes. Median Geriatric Depression Scale (GDS-15) scores were 3 (0–13) and 5 (0–13) respectively, indicating low levels of depressive symptoms.

Over a 12-month period, 3191 exercise groups were delivered. Mean number of groups in the residential homes was 82 and 78 in the nursing homes. Number of attendances at group exercise was 11,534/21,292 (54.2%) and 3295/6436 (51.2%) respectively.

Linear regression analysis revealed that depression, social engagement, and socio-economic characteristics were significantly associated with participant attendance at exercise groups in the residential homes, but none of these factors predicted attendance at group exercise in nursing homes.

**Conclusions:**

Older people living in long-term care are receptive to participating in exercise programmes, but there are individual and home-level reasons for attendance and non-attendance.

## Background

The ageing population of the United Kingdom (UK) continues to grow; whereas 40 years ago, life expectancy for men was 68 years, it has now risen to 78 years; for women, from 75 years to 82 years [1]. In England alone, the number of people aged over 65 has doubled since the early 1930’s. These figures have the potential to impact on demand for places in long-term care (LTC). Currently there are 19,000 LTC facilities in England, with a capacity of 441,000 places. Projections of future demand for residential care suggest that by 2020, this figure will have risen to 500,000 [2].

The importance of physical activity (PA) in this older population cannot be underestimated and there is good evidence that prevention or minimisation of the impact of sedentary lifestyles can have dramatic effects on physical and psychological health [3,4]. This is important given the global burden of multiple comorbidity, disability and frailty which is linked to decreased functional ability, performance of activities of daily living (ADLs), reduced health-related quality of life, all-cause mortality and costs associated with both health and social care services [3,5,6].

The American College of Sports Medicine (ACSM) [7] guidelines recommend that older adults (defined as those over 65 years old) should undertake 30 minutes of moderate intensity, aerobic exercise or activity, five times per week to incur any health benefits. Yet, globally and more specifically across the UK, the number of older people actually fulfilling this requirement is very small and is likely to be even lower amongst those resident in LTC [8] [9].

In 2010, a systematic review [8] of 49 randomised controlled trials (RCTs) investigated the effectiveness of physical rehabilitation in older adults resident in LTC, specifically assessing factors such as activity restriction, and strength and balance for older people. Thirty-three of the included trials found significant benefits of physical rehabilitation on outcomes of mobility, strength and ADLs. Importantly, physical activity was acceptable to those living in LTC.

Exercise was an intervention component in 46 of these 49 trials. In 27 studies, exercise was delivered in groups, with the mean attendance rate at group exercise reported in 17 studies as being 84% (range 71% – 97%). However, only four of the 49 trials were conducted in a UK setting. The overall mean sample size per clinical trial was 74 patients, and only nine trials included a sample ≥100. The authors concluded that larger scale studies with longer-term follow up were required in this understudied population.

Another systematic review [6] examined the motivators and barriers to activity amongst the oldest old, defined as aged over 80 years. From 44 qualitative and quantitative papers, a total of 61 motivators and 59 barriers to activity were identified. Although designed specifically to study those aged over 80 years, none of the 44 included papers exclusively described a sample of over 80 year olds; highlighting the lack of studies conducted involving adults in this age group. Additionally, only two of the studies were based in LTC (USA and Canada). However, from these two studies, the main barriers to physical activity were health or physical impairments [10]. Motivators often included physical/health benefits, having less pain, previous physical activity experiences and the social component and support of participating in physical activity [10,11].

Similar themes of physical and social benefits, family and staff support and previous lifestyle were identified as motivators to exercise class participation in a qualitative study of residents living in ‘low-level’ residential care; with health limitations including past medical conditions, pain, fear, lack of motivation and depression as barriers to exercise [12].

Since many previous studies identifying the barriers and motivators to exercise have been small and based in community settings, it is clear that more research is needed to identify predictors of attendance to exercise in institutionalised older people.

Existing research has focused upon the influence of patient or individual factors such as physical health, depression etc. Yet institutional or home level factors may also play a role in understanding barriers and motivators to participation.

There are a number of sociological models that provide a framework for analysing behavioural change. Often considered the most comprehensive model to account for exercise behaviour in older adults, social cognitive theory incorporating a social-ecological model i.e. a model to understand the relationship between personal and environmental factors, is the theoretical basis for adopting a programme of physical activity [13]. Behaviour is thought to be influenced by interacting and potentially confounding variables, such as those described above and can be categorised into intrapersonal, interpersonal, institutional/organisational, public policy and environmental factors and are strongly linked to self-efficacy i.e. a person’s sense of confidence or judgement in their ability to perform or accomplish a particular task or level of performance [14-16].

Using a social-ecological model, the aim of this study is to determine individual and ‘home level’ predictors of attendance at physiotherapy led exercise groups delivered in LTC across the UK. To achieve this, individual, clinical, and socio-demographic resident variables, and socio-economic characteristics of residential and nursing homes were identified and examined separately in order to predict exercise behaviours (specifically attendance at physiotherapy-led group exercise) of a large sample of older people resident in the different types of LTC who were recruited to a clinical trial [17].

These individual and ‘home level’ variables were selected, based on a comprehensive literature review, combined with the data available from the OPERA study and can be categorised as intrapersonal, interpersonal, institutional/organisational, and environmental factors acting as barriers and/or motivators to exercise [6,12,14,15]. This study is novel in that, although similar barriers and motivators have been explored previously as predictors of attendance to exercise, our study investigates a large cohort of older, frail adults resident in UK LTC facilities.

## Methods

### Study design

This was a nested cohort study using data from a large cluster-randomised controlled trial: Older People's Exercise in Residential and nursing Accommodation (OPERA). The rationale and methods for the OPERA trial have been reported in full elsewhere [17,18]. In brief, the trial included 1023 participants, aged over 65 years, living in 78 residential and nursing homes across Coventry and Warwickshire and North East London. Homes were randomised to either an active intervention (including staff depression awareness training and a whole home physical activation and exercise programme) or control (staff depression awareness training only). Follow up of the study participants was undertaken at 12 months post-randomisation; only data from the active intervention arm were used for this analysis.

### Participants

A subsample of 428 adults aged over 75 years residing in 34 homes randomised to deliver exercise was identified. Although 35 intervention homes were randomised, one had 0% attendance because no residents were actually eligible to participate in the exercise groups. This home was given staff depression awareness training and due to the principles of intention to treat, it was analysed in the intervention arm of the original OPERA study. However, for this regression analysis, as none of the residents were eligible to attend exercise, data i.e. predictors for attendance, were not included.

All participants aged over 75 were selected in this analysis because of lack of research investigating the ‘old’ to ‘oldest old’ living in LTC [6,19]. No upper age limit was applied.

### Data collection

Research nurses undertook baseline assessments on home residents. These took approximately one hour to complete for each participant. Questionnaire instruments included: the Geriatric Depression Scale (GDS-15), EuroQol (EQ-5D), Mini Mental State Examination (MMSE), fear of falling (yes/no) and a Short Physical Performance Battery (SPPB) to measure lower limb function [18,20]. The resident’s key worker, or home manager, completed a proxy EQ-5D, Barthel Index and social engagement scale (MDS). Demographic and socioeconomic data were collected from individual care plans for each participant. The home manager was asked to provide institutional level data, including size of home, number of beds and residents, activities already delivered within the home and whether they employed an activities co-ordinator [18].

Before the group exercise intervention commenced, the physiotherapists also undertook individual assessments of each resident to ascertain their baseline level of physical activity. This included determining eligibility to take part in exercise sessions and other considerations such as pain status, visual acuity, hearing, communication issues, and cognitive impairment. Functional ability was evaluated by assessment of sitting and standing ability, ability to rise from a chair, mobility, use of walking aids and an estimate of the baseline physical activity levels i.e. approximate amount of time spent walking/being active in a normal day.

Ethical review for the study was provided by the Joint University College London/University College London Hospital Committees on the Ethics of Human Research (Committee A), now known as Central London REC 4 (REC reference 07/Q0505/56).

### Exercise Intervention

Physiotherapists responsible for delivery of the intervention received an in depth two day training programme. This involved teaching on the processes and rationale of the trial as well as instruction on how to deliver exercise groups. Each physiotherapist was assigned to certain homes and liaised with the home manager/senior carer to arrange suitable times for running the exercise groups.

Individual exercise intensity for each participant was determined based on their initial assessment, beginning at a specific level; but which could be progressed:

- Level 1: Chair based lower level intensity aerobic and strength exercises.
- Level 2: Moderate intensity exercises performed in sitting and/or supported standing.
- Level 3: Moderate to high intensity exercises performed more dynamically in sitting and standing and incorporating walking and dancing activities.

Delivered by the physiotherapist, group exercise sessions ran twice weekly for 12 months. Set to appropriate music, groups consisted of a 5–10 minute warm up, a progressive resistance exercise section lasting approximately 15 minutes, where, based on the results of individual assessment, participants used soft hand weights. These weights ranged initially from 200 g to 1.0 kg and ankle weights from 0.5 kg to 1.5 kg. This was followed by a 15–20 minute moderate intensity aerobic section and a 5–10 minute cool down.

However, in general, the majority of groups were delivered as Level 1 groups due to the needs of the participants and to ensure safety, as often the groups were large and delivered solely by the physiotherapist. In some instances, however, mixed level groups were run i.e. some residents stood to perform Level 2 exercises whilst others continued at Level 1. This was only possible if a member of staff from the home was available to assist the physiotherapist.

As the intervention was designed as a whole home approach, all residents, including non-study participants could potentially be exposed to the intervention. Consenting to attend the class was done informally, reflecting best clinical practice. All residents were encouraged to attend by the physiotherapists or home staff, who gave them a brief description of the exercise intervention at the beginning of each session. Residents could then give consent and participate, or refuse to participate by leaving the room, or request to leave the room or not do the exercises [21]. Only data from those who consented to take part in the OPERA study have been included in this analysis.

### Outcome variables

Percentage attendance at the exercise groups was calculated for each home and for each individual participant.

$$ \begin{array}{l} Percentage\  attend ance\ \\ {} = \frac{Number\  of\  groups\  actually\  attend ed}{Number\  of\  possible\  groups\  available\ to\  attend}\times 100\end{array} $$

This calculation was used because a different number of exercise groups ran in each home and therefore, percentage attendance allowed a standardised approach to capture attendance.

Data on maximum possible attendance was calculated for each home by multiplying the number of study participants by the number of groups delivered. However, in practice, actual ‘exposure’ to group exercise varied on an individual level because of deaths, transfers to other institutions or inability to attend due to illness/other reason. Data were recorded prospectively to allow accurate estimation of individual attendance.

### Predictor variables

Of the variables collected by the OPERA research team, nine baseline individual, clinical and socio-demographic variables, and socio-economic characteristics of residential and nursing homes were selected as potential predictors of attendance to the exercise groups.

### Number of chronic conditions

Health limitations and impairments including co-morbidities/chronic conditions are amongst the most common reasons influencing attendance to exercise [6,10,12]. The original study team devised a pre-determined, *a priori* list of common medical conditions informed using existing co-morbidity scales (cancer, stroke, dementia, depression, anxiety, osteoporosis, chronic lung disease, urinary incontinence) [21]. Co-morbidity data were extracted from participant care plans i.e. number of co-morbid conditions was used in the analysis.

### Depression

Depression is a known barrier to attendance at exercise [12]. Prevalence of depression was the primary outcome of the OPERA study and depression is more common in older people living in LTC than those residing in their own homes [22,23]. The validated 15 item short form Geriatric Depression Scale (GDS) was selected because it was specifically developed for use with older people and has been well validated in residential situations [24]. The following scoring guidelines were used: 0 – 5 no depression, 6 – 10 mild depression and 11 – 15 indicative of more severe depression [18,25]. However, not all residents could answer all 15 questions, therefore a cut point was used; the GDS score was valid if the resident could answer ≥ 10 of the 15 questions [26].

### Lower limb function

Lower limb function, associated mobility and functional ability have an important influence on participation in physical activity [27]. The Short Physical Performance Battery (SPPB) measures lower limb function; it incorporates balance tests, a four metre timed walk and time to stand from a chair exercise. A score is given for performance in each category, ranging from 0–4, with four indicating the highest level of performance (scale range 0–12). The measure can be used to characterise physical functional status and has been shown to predict mortality and institutionalisation [20].

### Fear of falling

Fear of falling can have an influence on attendance to exercise groups in LTC [10,28]. Participants were asked a simple yes/no question about fear of falling [18] .

### Social Engagement

The social aspect of group exercise can be a very important interpersonal factor influencing participation in exercise/PA [29]. A proxy psychosocial well-being measure, taken from the Minimum Data Set (MDS) [30] was used to collect information regarding participants’ interaction with others and whether they took part in activities in the home. The MDS is an American assessment system used in care planning in nursing homes and for research purposes. The OPERA research nurses asked care home staff to indicate (yes/no) whether six specific aspects of the MDS were applicable to each participant. For this analysis, two of these six elements were relevant, “initiates interaction with others” and “pursues involvement in the life of the facility”. These items were selected because they linked to self-efficacy; thus examined the participants’ active involvement in social aspects of the home, whereas other items in the full scale were not relevant for this study.

### Activity co-ordinator

Previous experiences of exercise and physical activity can influence attendance [12,28]. If residents were already undertaking regular exercise and other activities prior to the OPERA study and these activities were organised and/or led by an established activity co-ordinator (organisational/institutional factor), this could influence their participation in the exercise groups. Each home manager identified, at baseline, whether an activities co-ordinator was employed in each home (yes or no). This was used as an indicator of accessibility to exercise and/or PA.

### Home-level socioeconomic status

There is evidence to suggest that attendance and participation in exercise is influenced by socio-economic status [31]. Economically disadvantaged individuals are less likely to engage with exercise/physical activity interventions [32] therefore, funding status of the home was included as a crude proxy indicator of socio-economic status (SES) of residents within the home. This was defined as residents either fully self-funding their own care or receiving financial state support (partial or full) from Social Services.

### Statistical analysis

In the UK, older people requiring LTC are assessed for their physical and mental health needs. Their level of need determines whether a placement in residential or nursing care is more suitable. Residential homes provide short (respite) or long-term care and offer support with personal care only e.g. washing, dressing and provision of medications. Nursing homes offer the same help with personal care but also have a qualified nurse on duty 24 hours a day to provide nursing care for residents with more complex health needs. Given that resident characteristics in the two types of home were likely to differ, we conducted all analyses separately, based on type of accommodation i.e. residential and nursing.

Descriptive analyses were undertaken on all variables at baseline and at 12 months where appropriate. Continuous data were analysed using mean and standard deviation (SD) or median (range) for skewed data.

Subsequently, univariate and multivariate linear regression analyses were conducted to identify potential predictors of attendance. Following the univariate analysis of the individual variables and environmental variables, a stepwise modelling approach was implemented, using all of the variables of interest i.e. both significant and non-significant variables from the univariate analysis.

The individual intra and interpersonal variables relating to physical and psychological health were added to the multivariate model first: (1) number of chronic conditions, (2) GDS score, (3) SPPB score (4) fear of falling, (5) initiates interaction with others (6) pursues involvement in the life of the facility.

In a separate multivariate analysis, the organisational/socio-economic variables were added in the following order: (1) activity co-ordinator employed in the home (2) fully self-funding care (3) partial or full Social Services funding.

## Results

### Home Characteristics

The 34 homes randomised to deliver exercise comprised residential (n = 25) and nursing homes (n = 9). Dementia specialist care was also integrated within two residential (n = 2) and one nursing (n = 1) home. Twenty seven (79%) of the intervention homes were privately owned and seven (21%) were run as voluntary or charity homes. The number of beds in homes ranged from 17–62 with the number of permanent residents ranging from 13–61. Activity co-ordinators were employed in 17/25 (68%) residential homes and 7/9 (78%) nursing homes.

### Participant characteristics

Baseline variables and characteristics of the 428 recruited participants (n = 326 in residential homes and n = 102 in nursing homes) are described in Table 1. At baseline mean age (SD) of those in residential homes was 88.3 (6.0) years and 87.0 (6.1) years in nursing homes and 77% and 72% were female, respectively. Baseline cognitive function was found to differ by home status: the median (range) MMSE score was 19 (0–30) for those in residential homes compared to 14 (0–29) for those in nursing homes, although both are within the range suggesting moderate cognitive impairment. Measured with the GDS-15, valid depression scores were median (range) of 3 (0–13) and 5 (0–13) in residential and nursing homes respectively. More detailed analysis of the participants showed that, in the residential homes, 69% of residents scored 0–5 on the GDS, 27% scored 6–10 and 4% scored 11–15. In the nursing homes, 70% of residents scored 0–5 on the GDS, 24% scored 6–10 and 6% scored 11–15.

**Table 1:** **Participant and home characteristics (Baseline and 12 months)**

	**Residential homes (n = 25)**		**Nursing homes (n = 9)**	
	**Baseline (n = 326)**	**12 months (n = 230)**	**Baseline (n = 102)**	**12 months (n = 72)**
**Age** mean (sd)	88.3 (6.0)	88.6 (6.0)	87.0 (6.1)	89.0 (5.7)
**Gender**				
Male (%)	75 (23.0) 251	59 (25.7) 171	29 (28.4) 73	20 (27.8) 52
Female (%)	(77.0)	(74.3)	(71.6)	(72.2)
**Cognitive function (MMSE, 0–30)**				
Median (range)	19 (0–30)	18 (0–30)	14 (0–29)	13 (0–30)
**No. of co-morbidities**				
Median (range)	2 (0–6)	Not available	3 (0–7)	Not available
**Depression (GDS-15)**				
Median (range)	3 (0–13)	3 (0–13)	5 (0–13)	5 (0–12)
Missing (n)	17	27	15	13
**Breakdown of scores:**				
0–5	69%	76%	70%	61%
6–10	27%	20%	24%	36%
11-15	4%	4%	6%	3%
**Lower limb function (SPPB, 0–12)**				
Median total SPPB score (range)	1 (0–10)	1 (0–10)	0 (0–8)	0 (0–8)
**Fear of falling**				
Yes (%)	134 (41.1)	75 (32.6)	35 (34.3)	18 (25.0)
No (%)	170 (52.1)	118 (51.3)	53 (52.0)	38 (52.8)
Don’t know (%)	1 (0.3)	9 (3.9)	6 (5.9)	1 (1.4)
Missing (%)	21 (6.4)	28 (12.2)	8 (7.8)	15 (20.8)
**Social engagement Initiates interaction with others**				
Yes (%)	236 (72.4)	153 (66.5)	76 (74.5)	50 (69.4)
No (%)	76 (23.3)	71 (30.9)	26 (25.5)	22 (30.6)
Missing (%)	14 (4.3)	6 (2.6)	0	0
**Pursues involvement in the life of the facility**				
Yes (%)	225 (69.0)	133 (57.8)	66 (64.7)	46 (63.9)
No (%)	87 (26.7)	93 (40.4)	35 (34.3)	26 (36.1)
Missing (%)	14 (4.3)	4 (1.7)	1 (1.0)	0
**Activity co-ordinator**				
Yes (%)	17 (68.0)	Not available	7 (77.8)	Not available
No (%)	8 (32.0)		2 (22.2)	
**Socio-economic status**				
Fully self-funded (%) Social Services	169 (51.8)	Not available	24 (23.5)	Not available
funded (%) Partially funded by social	94 (28.8)		71 (69.6)	
services (%)	39 (12.0) 24		5 (4.9)	
Missing (%)	(7.4)		2 (2.0)	

At 12 months, data from 302 (71%) participants (n = 230 in residential homes and n = 72 in nursing homes) were available (Table 1). MMSE scores reduced by 1 point in both the residential homes, 14 (0–29), and nursing homes, 13 (0–30), but median GDS scores remained the same. However, distribution of scores varied over the follow up period: 76% of residents scored 0–5, 20% scored 6–10 and 4% scored 11–15 in the residential homes and 61% of residents scored 0–5, 36% scored 6–10 and 3% scored 11–15 in the nursing homes.

SPPB scores were very low. Overall baseline median (range) scores were 1 (0–10) and 0 (0–8) in residential and nursing homes respectively with no changes over 12 months. Looking closer at the three components of the test, 57% of residential home participants were unable to score on the balance test, 34% unable to complete the 4-metre walking test and 88% unable to complete the chair stand test. In the nursing homes, 69% of participants were unable to score on the balance test, 58% on the 4-metre walking test and 90% on the chair stand test. Thus, baseline lower limb function was significantly worse in nursing home residents.

### Attendance at the Exercise Groups

Over a 12-month period, 3,191 exercise groups were delivered to home residents. This ranged from 69 to 95 exercise sessions delivered to those in residential homes and 67 to 87 exercise sessions to those in the nursing homes. The number of possible attendances available was calculated (excluding those who had died, moved or were ineligible to take part in the groups), resulting in 21,292 possible attendances in residential homes and 6,436 attendances in the nursing homes. Of these, the actual attendance rate at group exercise was 11,534 (54.2%) in the 25 residential homes and 3,295 (51.2%) in the nine nursing homes included in this analysis.

### Linear Regression Analysis

#### Residential Homes

Although only 31% of the residential home sample had depressive symptoms at baseline, univariate analysis revealed that depression scores were independently and significantly associated with reduced participant attendance at the exercise sessions (GDS −2.95, 95% CI −4.21 to −1.68; p <0.001) (Table 2). This suggests that for every one-unit increase in GDS score, it reduced percentage attendance at group exercise by 3%.

**Table 2:** **Univariate analysis of baseline variables and relationship with attendance at residential home exercise sessions**

**Variable**	**n**	**Relationship with % attendance at group exercise**			
		**Unstandardised regression coefficients (95% CI)**	**R** ^**2**^	**Standardised coefficients (β)**	**P-value**
Number of chronic conditions^0^	326	−2.54 (−5.75 to 0.67)	0.007	−0.09	0.121
GDS score^1^	309	−2.95 (−4.21 to −1.68)	0.064	−0.25	<0.001
Total SPPB score	326	1.39 (−0.33 to 3.10)	0.008	0.09	0.112
Fear of falling^2^	305	0.20 (−0.49 to 0.89)	0.001	0.03	0.567
Initiates interaction with others^3^	313	11.84 (3.03 to 20.65)	0.022	0.15	0.009
Pursues involvement in the life of the facility^3^	313	16.42 (8.09 to 24.74)	0.046	0.22	<0.001
Activities co-ordinator employed in the home	326	9.13 (1.07 to 17.19)	0.02	0.12	0.026
**Home level socio-economics:**					
Self-funding	274	0.50 (0.09 to 0.91)	0.20	0.14	0.018
Social services funded	274	−0.35 (−0.63 to −0.08)	0.02	−0.15	0.012

^0^Medical conditions taken from resident care plans.

^1^GDS score is valid if the resident could answer ≥ 10 of the 15 questions.

^2^Self-reported.

^3^Proxy reports from care staff.

Two social engagement measures from the MDS were significantly associated with attendance at group exercise: initiates interaction with others (MDS 11.84, 95% CI 3.03 to 20.65; p = 0.009) and pursues involvement in the life of the facility (MDS 16.42, 95% CI 8.09 to 27.74; p = <0.001).

From the organisational/institutional variables, the presence of an activity co-ordinator (9.13, 95% CI 1.07 to 17.19; p = 0.026) and being self-funded were also predictive of attendance at group exercise (0.50 95% CI 0.09 to 0.91; p = 0.018). Conversely, care being fully funded by Social Services (−0.35 95% CI −0.63 to −0.08; p = 0.012) was negatively associated with attendance at group exercise in the residential homes.

In the multivariate model (Table 3), one individual interpersonal and one intrapersonal variable was significantly associated with attendance to the residential home exercise groups (one negatively and one positively). Residents with depressive symptoms were significantly less likely to attend (−2.41 95% CI −3.78 to −1.03; p = 0.001). Those who scored ‘yes’ on “pursuing involvement in the life of the facility” were significantly more likely to attend exercise groups (13.21, 95%CI 3.01 to 23.42; p = 0.011). However, the percentage of the response variable variation that is explained by the linear model or R^2^ value should be considered as it is only 8.7%.

**Table 3:** **Multivariate analysis of individual variables of participants in residential homes with % attendance as the dependent variable**

**Explanatory variables**	**Unstandardised coefficients (95% CI)**	**Standardised coefficients (β)**	**P-value**
Valid GDS score	−2.41 (−3.78 to −1.03)	−0.21	0.001
Pursues involvement in the life of the facility	13.21 (3.01 to 23.42)	0.17	0.011
Constant	27.88 (6.53 to 49.24)		
R^2^ = 8.7%^1^			

^1^Proportion of variance explained by the model.

#### Nursing Homes

No statistically significant associations were found between any predictor ande at the exercise groups in either univariate or multivariate linear regression analysis of the nursing home data (Table 4.)

**Table 4:** **Univariate analysis of baseline variables and relationship with attendance at nursing home exercise sessions**

**Variable**	**n**	**Relationship with percentage attendance at group exercise**			
		**Unstandardised regression coefficients (95% CI)**	**R** ^**2**^	**Standardised coefficients (β)**	**P-value**
Number of chronic conditions^0^	102	−1.68 (−6.93 to 3.57)	0.004	−0.06	0.526
GDS score^1^	87	−0.20 (−2.67 to 2.28)	0.000	−0.02	0.875
Total SPPB score	102	0.97 (−2.84 to 4.77)	0.003	0.05	0.616
Fear of falling^2^	94	0.02 (−0.29 to 0.33)	0.000	0.01	0.892
Initiates interaction with others^3^	102	6.63 (−9.74 to 23.0)	0.006	0.08	0.424
Pursues involvement in the life of the facility^3^	101	10.4 (−4.63 to 25.46)	0.019	0.14	0.173
Activities co-ordinator employed in the home	102	−14.1 (−31.88 to 3.73)	0.024	−0.16	0.120
**Home level socio-economics**					
Self-funding	69	0.04 (−1.25 to 1.33)	0.000	0.01	0.955
Social services funded	69	−0.48 (−1.54 to 0.58)	0.012	−0.11	0.367

^0^Medical conditions taken from resident care plans.

^1^GDS score is valid if the resident could answer ≥ 10 of the 15 questions.

^2^Self-reported.

^3^Proxy reports from care staff.

## Discussion

This study used a prospective cohort design to investigate individual and environmental/organisational factors as predictors of attendance to group exercise offered to an older population resident in LTC. We used an efficient nested cohort study design to select adults aged 75 to 107 years, participating in a cluster-RCT of a physical activation programme which included group exercise versus a control intervention incorporating depression awareness training for care home staff. This is the first large study of exercise adherence within residential and nursing homes in the UK.

### Attendance

Overall percentage attendance to the exercise groups in the residential and nursing homes was just over 50% in both settings. For the most part this is slightly lower than in previous trials where attendance rates to group exercise in LTC has ranged from 42.5% to 100% [28]. In the process evaluation of the OPERA study, Ellard et al. [33] conclude that this was a low level of attendance, particularly amongst those who were depressed, partly due to issues such as frailty, cognitive impairment and lack of staff time to assist in getting residents to the groups. Thus, overall exposure to adequate intensity of exercise was low, subsequently resulting in an overall negative effect of the intervention on depression [21].

However, for residents attending regularly, continued attendance may have been due to the physiological and psychological beneficial effects of exercise [28]. Potentially, participants profiting from exercise or even perceiving that they are benefitting will have attended more groups, compared to those perceiving no benefit. Plausibly, therefore, although short term, these improvements in physical and psychological well-being and feelings of enjoyment and achievement linked to self-efficacy and mastery may have encouraged attendance [15,16].

It is also argued that living in LTC can be a passive experience, typified by increasing dependency and lack of control. However, exercise interventions similar to that used in OPERA have been shown successfully to give participants the opportunity to regain some control and sense of self-worth [10] and therefore may have been another possible explanation for participant attendance. The staff and residents in the intervention homes were very positive about the exercise groups and during observations undertaken during the process evaluation, the exercise intervention did appear to make a short term difference to residents’ mood and physical abilities [33].

### Predictors of attendance

Of the nine individual and home level characteristics variables investigated in this study, six were associated with group attendance in univariate analysis in the residential home population, four having a positive effect, and two, a negative effect. However, after adjustment, only two variables were associated with attendance in the residential homes. None of the predictor variables were significantly associated in either univariate or multivariate analysis with attendance in the nursing homes.

Despite health status/physical limitations including pain, fear of falling and number of chronic conditions being common barriers and motivators to exercise and physical activity [6,10-12], number of co-morbidities as a measure of health status was not significantly predictive of attendance in LTC. Potentially this was because all residents had co-morbidities and there was lack of variation amongst this age group. However, this predictor is important, because health issues have consistently been shown to be a common barrier and motivator to attendance at exercise and number of illnesses, although a crude indicator on its own, may be a significant indicator of frailty. But perhaps frailty as a distinct health state is crucial, and needs to be considered as more than just number of health problems.

In our study, a large proportion of our sample would have been classified as ‘frail older adults’ based on their SPPB results for lower limb function, i.e. median baseline scores and 12 month follow up scores were zero in nursing homes and one in residential homes. Frailty, including symptoms of muscle weakness, slow walking speed and low physical activity [34] can result in non-compliance and low participation in physical activity and structured exercise programmes. This is common amongst older people and even more so those resident in LTC [8,10,35].

Despite this, at initial assessment, lower limb function was not significantly associated with attendance at the exercise groups. However, it could be argued that the SPPB was an inappropriate measure to use in this population, as it was originally designed for community dwelling adults and lacks sensitivity in this particular group. This is highlighted by the obvious floor effect in this current sample [21].

Thirdly, fear of falling was not associated with attendance at the exercise groups. Numbers of participants reporting fear of falling was lower than anticipated, possibly because of the lack of activity being undertaken by some residents i.e. they were not fearful of falling because they were not active to the extent that falling might be a risk or concern. Instead, and in line with the idea of passivity, participants were moved around the homes and often brought to groups in wheelchairs [10]. Interestingly, fear of falling declined over time. Alongside this, residents may not have been fearful because they know that carers are nearby should they need help, compared to older people living alone in the community who may not have anyone close by to help them if they do fall.

Although data shows that two in every five older people living in care are depressed [36], the negative effect of elevated depression scores (suggesting more depressive symptoms) were only predictive of attendance to the exercise groups in the residential home sample. This is reiterated by studies reporting that depressed older people are less likely to attend or participate in exercise [33,35]. An implication of this is that those engaged in the life of the home would be more likely to join in with group exercise. However, exercise groups do not necessarily attract everybody, and an alternative approach to engage those with depression in exercise may be beneficial. Additionally, early detection, diagnosis and treatment of depression in this population is vital.

Although the depression scores of the nursing home residents were indicative of more depressive symptoms, depression was not significantly associated with attendance at the exercise groups in the nursing homes. These results may have been due to the smaller sample size within the nursing home population or may be due to selection bias. For example, GDS scores were skewed due to the inability of some residents to complete all 15 GDS test questions due to cognitive impairment; therefore, a smaller number or lower proportion (n = 87) of actual valid GDS scores were available for analysis. This again implies that the GDS was perhaps not the most appropriate measure to capture depression in this vulnerable population [37].

Social engagement and support from others e.g. initiating interaction with other residents and pursuing involvement in the life of the facility can be related to self-efficacy or the residents’ beliefs in their own ability to complete tasks and achieve goals. As in this study, socialising, interaction and support from other residents, carers and family have previously been found to have a positive effect on attendance to physical activity in community dwelling older adults and those living in low level residential care [12,29,38].

However, the two social engagement measures were not found to predict attendance at group exercise in the nursing homes. The MDS tool was designed to be used as a direct observational tool [21] but in this instance it was used as a proxy measure based upon staff judgement rather than actual observation of social involvement or extent of engagement with others. However, due to the large sample size, it was not possible to observe individual study participants. Staff from homes were asked to complete data collection forms on social engagement for all participants, but this process was often rushed, due to time constraints and staffing issues. However, there was no evidence to suggest differential error (measurement bias) between nursing and residential homes, but we cannot exclude the possibility that the data may not have been always entirely accurate.

Socioeconomic status underlies major determinants of health including health behaviour. Measured by income, education, or occupation, SES has been associated with a wide range of health conditions, including cardiovascular disease, arthritis and diabetes. This is evident in economically deprived groups where engagement with activity interventions and exercise and physical activity levels are consistently lower [32]. Residents’ SES was a significant predictor of attendance to group exercise but only in the univariate analysis of the residential home sample. This might be explained by the differences in funding; the proportion of fully self-funded participants and therefore, potentially less economically deprived, was double in residential homes (52%) compared to that in the nursing homes (24%).

Similarly, the presence of an activity co-ordinator was only a significant predictor of attendance to group exercise in the residential home sample. Ellard et al. [33] considered the negative effect of the exercise intervention in terms of whether the intervention changed the culture of physical activity within homes. They report that whilst some activity co-ordinators were observed just performing their own role, many were also assigned other tasks e.g. personal care, helping with meals etc. therefore taking them away from their primary activity role. Thus it is possible that homes with no activity co-ordinator or those spending less time involved in activities may have had lower levels of attendance as the residents were less used to activities of any kind, let alone physical activity. Alternatively, the influence of a motivated, enthusiastic activity co-ordinator may have impacted positively on attendance [39].

The differences in results between the residential and nursing homes should be interpreted with caution. Despite the overall large sample size, the number of residents completing the intervention in the nine nursing homes was relatively small (n = 72) resulting in lower statistical power in this group. Therefore, the lack of any significant results from the nursing home data may be due to the size of the sample. Additionally, the low R^2^ value in the multivariate analysis indicates that the model may not be the best fit for the data. However, as in this instance, lower R^2^ values are often expected when predicting human behaviour and conclusions can still be made from the significant coefficients representing changes in the predictor values and their association with changes in the response values [40].

It is recognised that the older people participating in the study were from a target population, in selected geographical areas and those who gave consent were motivated to volunteer for a 12 month clinical trial involving exercise. For those whom assent was needed, family members may have given agreement for their relatives to participate when it was not particularly appropriate or vice versa.

Attrition rate, perhaps not surprisingly, was a limitation of the study. As expected, the mortality rate in this older population was high, with a total of 11% crude death rate over the 12 months. Additionally, although taken into consideration in the denominator, other uncontrollable reasons for non-attendance i.e. illness, transfers in and out of homes, and ineligibility for attendance at group exercise, resulted in over 6000 potential group attendances being lost. Therefore, this will affect percentage attendance figures and data that may have been incorporated in this analysis.

The primary aim of the trial was to investigate depression rather than predictors of attendance to group exercise, therefore other variables that may have contributed as predictors were not available for inclusion in this analysis i.e. previous exercise/physical activity levels or experience. Analysing the data by type of accommodation may have also had an impact on the results; this approach was taken because we anticipated a difference in resident characteristics within the homes. Therefore further work is needed to examine other factors, including type of accommodation, which may influence attendance to group exercise in this population.

Nevertheless, our findings are based upon a large sample of older adults up to the age of 107 years, incorporating high quality data from validated, standardised measures collected within the framework of a rigorously conducted, high quality trial. Despite the practical and methodological challenges of conducting clinical research with a very frail elderly population, a very large sample of participants from care homes across Coventry, Warwickshire and London were recruited. Our study provides good quality research findings on a vulnerable population resident in LTC in the UK.

## Conclusion

In our sample of adults aged 75 to 107 years, predictors of attendance to group exercise included lower depression scores, perceived social support and active involvement in the home and their influence on self-efficacy and home-level socio-demographics and environmental constraints, but only in residential homes. Self-funding residents within residential homes that employ an activities co-ordinator are more likely to attend group exercise classes; and individual characteristics, such as physical health, lower limb strength and fear of falling were not found to predict attendance at group exercise. None of our selected variables were predictive of attendance to group exercise in our smaller sample of nursing homes.

Awareness of the factors associated with attendance and non-attendance to exercise may help health professionals and care staff working in LTC to overcome some of the barriers and negative perceptions that older people have toward exercise and physical activity.

The results indicate that older people, even those who are frail with high levels of dependency, living in LTC, are prepared to participate in interventions involving physical activity. This study has helped to clarify that certain intrapersonal, interpersonal and environmental factors can predict attendance to group exercise in the long-term care setting.

## Abbreviations

UK: United Kingdom
LTC: Long-term Care
PA: Physical Activity
ADLs: Activities of Daily Living
ACSM: American College of Sports Medicine
RCT: Randomised Controlled Trial
OPERA: Older People’s Exercise in Residential and nursing Accommodation
GDS: Geriatric Depression Scale
MMSE: Mini Mental State Examination
SPPB: Short Physical Performance Battery
MDS: Minimum Data Set
SES: Socio-economic Status
SD: Standard Deviation
